# Identification and Validation of the lncRNA BACE1-AS as Immune-Related Influencing Factor in Tumorigenesis following Pan-Carcinoma Analysis

**DOI:** 10.1155/2021/1589864

**Published:** 2021-12-08

**Authors:** Mengmeng Wang, Di Chen, Yushuang Xu, Mengjun Qiu, Xin Jiang, Zhifan Xiong

**Affiliations:** Division of Gastroenterology, Liyuan Hospital, Tongji Medical College, Huazhong University of Science and Technology, Wuhan 430077, China

## Abstract

**Background:**

The lncRNA BACE1-AS was identified as a plasma molecular marker in the early diagnosis of Alzheimer's disease, but its role in tumors remains poorly defined.

**Methods:**

The expression patterns, genomic mutation, and prognostic significance of BACE1-AS in pan-cancers were compared by analyzing 32 types of tumors from The Cancer Genome Atlas and cBioPortal databases. The relationships between BACE1-AS expression levels and the degree of immune cell infiltration, immune components, and immune-related genes were explored. The possible molecular mechanisms of BACE1-AS in tumors were explored using gene set enrichment analysis (GSEA). Finally, the role of BACE1-AS in hepatocellular carcinoma was confirmed via quantitative real-time polymerase chain reaction (qRT-PCR).

**Results:**

BACE1-AS expression levels were significantly upregulated in LIHC, GBM, KIRC, CHOL, STAD, KICH, COAD, and PRAD. Higher expression levels of BACE1-AS were associated with worse overall survival in patients with HNSC and LIHC, while the opposite was found in PCPG and THCA. The overall mutation rate of BACE1-AS in pan-cancer was only approximately 0.9%, and it occurred mainly in uveal melanoma and uterine carcinoma. Generally, BACE1-AS expression was negatively correlated with the immune microenvironment. BACE1-AS expression was mainly related to naïve B cells, activated memory CD4 T cells, monocytes, M1 macrophages, M2 macrophages, and resting mast cells. The potential mechanisms of BACE1-AS in tumors were mainly via regulating the activities of B cell-mediated immunity, immune response regulating cell surface receptor signaling, RNA binding in posttranscriptional gene silencing, B cell receptor signaling pathways, and immune receptor activity. Finally, the qRT-PCR results confirmed that the expression levels of BACE1-AS in hepatocellular carcinoma cell lines were upregulated.

**Conclusions:**

Overall, our results suggest that BACE1-AS is associated with the expression, prognosis, and rate of immune cell infiltration of most tumors. Thus, BACE1-AS may be a potential target for immunotherapies aimed at improving cancer patient outcomes.

## 1. Introduction

Malignant tumors are a major global public health problem that seriously threatens human health, and their overall incidence is rising worldwide [1]. In many developed countries, cancer-related deaths are second only to heart disease. In China, the incidence and mortality of cancer are the highest globally [2]. Worldwide, the top 10 cancers are lung, breast, stomach, colorectal, liver, esophagus, cervical, thyroid, pancreatic, and bladder. On average, more than 6,000 people die of cancer daily. In fact, approximately five people die of cancer every minute [3]. Thus, malignant tumors are a problem that threatens the health of all humans: they have a complex cause, their treatments have the worse therapeutic effects, and their prognosis is poor.

The theory that the genesis of a tumor is primarily determined by its genetic material is based on the tumor-centric view. Traditional methods such as surgical resection, radiotherapy, and chemotherapy are all derived from this theory; these methods all inevitably have the same destructive effect on normal tissues [4]. Recently, tumor immunotherapy targeting the immune system has revolutionized cancer treatment as well as given a new perspective in the mechanisms underlying tumor pathogenesis and drug resistance [5]. In particular, the current use of the cytotoxic T lymphocyte-associated protein 4 (CTLA4) and programmed cell death-1 (PD-1)/programmed cell death-ligand 1 (PD-L1) has become one of the most promising immunotherapies [6]. However, the efficacy of tumor immunotherapy is limited by the high degree of tumor microenvironment (TME) immunosuppression and the low immunogenicity of cancer cells [7].

Long noncoding RNAs (lncRNAs), a group of RNAs with a length of more than 200 nucleotides, do not contain protein-coding transcripts due to the lack of an open reading frame [8]. However, they play a significant role in tumor proliferation, invasion, metastasis, and chemoradiotherapy resistance [9]. The lncRNA MALAT1, by mediating the secretion of basic fibroblast growth factor (FGF-2), can inhibit inflammatory cytokine release and promote the proliferation, migration, and invasion of FTC133 cells [10]. Cao et al. found that the lncRNA MM2P modulates M2 macrophage polarization and weakens M2-mediated neovascularization [11]. BACE1-AS is transcribed from the opposite strand of *β*-secretase 1 (BACE1) and can pair with BACE1 mRNA to change its spatial structure and increase its stability to promote protein translation in a positive feed-forward fashion [12, 13]. In other words, BACE1 and BACE1-AS can coregulate biological activities and processes and functionally interact with each other. BACE1 is a protease belonging to the *β*-secretase family and is thought to play an important role in Alzheimer's disease via the processing of amyloid precursor protein (APP) in neurotoxic A*β* peptides. Recently, BACE1 has been reported to be abnormally expressed in a variety of tumors and is associated with poor prognosis in colon cancer, non-small-cell lung cancer, invasive ductal carcinoma of the breast, glioblastoma, and gastric cancer [14–18].

Whether BACE1-AS has a similar effect in malignant tumors is unknown. Therefore, we conducted an in-depth analysis of 32 human tumors from The Cancer Genome Atlas (TCGA) database to further explore the role of BACE1-AS in tumors and its relationship with tumor immunity.

## 2. Materials and Methods

### 2.1. Pan-Cancer Data Source and Analysis

The expression matrix of BACE1-AS for 32 human tumors was obtained from TCGA database (https://portal.gdc.cancer.gov/), an open database covering 32 human cancer types, containing more than 30,000 tumor samples and information on the expression of more than 20,000 genes [19]. The mutations and copy number alterations (CNA) of BACE1-AS in pan-cancer were obtained by the cBioPortal for Cancer Genomics (http://www.cbioportal.org).

### 2.2. Kaplan-Meier Survival Analysis and Correlation between BACE1-AS Expression and Clinical Characteristics

The significance of BACE1-AS expression level for survival of 32 cancer types was achieved through R packages “survival” and “survminer” by Kaplan-Meier survival analysis. Overall survival (OS), disease-specific survival (DSS), progression-free interval (PFI), and disease-free interval (DFI) were analyzed to evaluate prognostic value. The log-rank test was used to evaluate the statistical significance between them, and *P* < 0.05 was considered statistically significant. The R “ggpubr” and “limma” packages were used to analyze the association between BACE1-AS expression and clinical characteristics including age, gender, and stage.

### 2.3. Mutation Analysis

Mutation data for 32 cancer types were downloaded by UCSC XENA (http://xena.ucsc.edu/), and tumor mutation load, the number of mutations per megabase, was calculated. The correlation between the expression of BACE1-AS and tumor mutation burden (TMB) or microsatellite instability (MSI) was calculated by the Spearman test. The results were represented by radar map drawn by the R “fmsb” package.

### 2.4. Correlation Analysis of BACE1-AS Expression and Tumor Microenvironment

The tumor microenvironment (TME), a special environment surrounding the presence of tumor cells, is composed of tumor stroma, adjacent cells, blood vessels, peripheral immune cells, and immune molecules [20]. The changes of these important components in the tumor microenvironment play a key role in tumor growth, invasion, metastasis, and immune tolerance. The R “estimation” package and “limma” package were used to analyze the association between BACE1-AS expression and tumor stromal or tumor immune cells. The higher the stromal scores and immune scores, the lower the purity of tumor cells.

### 2.5. Tumor-Infiltrating Immune Cell Analysis

The relative proportion of infiltrating immune cells in a pan-cancer patient was calculated by the CIBERSORT algorithm. The correlation between BACE1-AS expression and immune cells was analyzed by the Spearman correlation test. The R “ggpubr” package was used to analyze the correlation between 32 kinds of human tumors and 22 kinds of immune cells. The correlation between the expression of BACE1-AS and immune-related genes was performed by the R “reshape2” and the “RColorBrewer” packages.

### 2.6. Mechanism Analysis of BACE1-AS in Pan-Cancer

The potential mechanism of BACE1-AS in pan-cancer was mainly revealed by gene set enrichment analysis (GSEA) including Kyoto Encyclopedia of Genes and Genomes (KEGG) and Gene Ontology (GO) terms. The GSEA was performed by R “ggplot2,” “enrichplot,” “org.Hs.eg.db,” and “clusterProfiler” packages based on “c2.cp.kegg.v7.1.symbols.gmt gene set” and “c5.all.v7.1.symbols.gmt”. In order to obtain the normalized enrichment fraction, nominal (NOM) *P* < 0.05 and false discovery rate (FDR) < 0.05 were considered to be significantly enriched.

### 2.7. Validation of the Role of BACE1-AS in Hepatocellular Carcinoma

The prognostic value of BACE1-AS combined with coexpression immune-related checkpoint genes for hepatocellular carcinoma was analyzed, and a prognostic model was established by Cox regression analysis. Furthermore, the expression level of BACE1-AS in HCC was detected by real-time quantitative reverse transcription PCR (qRT-PCR) at cell level. The sensitivity analysis of the common compounds was performed by R “pRRophetic” package.

### 2.8. Statistical Analysis

OS, DFI, DSS, and PFI were analyzed through R packages (R version 4.1.0) “survival” and “survminer” by the Kaplan-Meier method. The correlations between BACE1-AS expression level and TMB, MSI, stromal, tumor-infiltrating immune cells, and immune marker sets were calculated by the Spearman test. The correlation between BACE1-AS expression and clinical characteristics by the sensitivity analysis of the common compounds was analyzed by the Wilcoxon test. All statistics were achieved by SPSS statistical software (version 25.0.0). *P* value < 0.05 was considered statistically significant.

## 3. Results

### 3.1. BACE1-AS Is Abnormally Expressed in 32 Human Tumors

**Figure 1:**
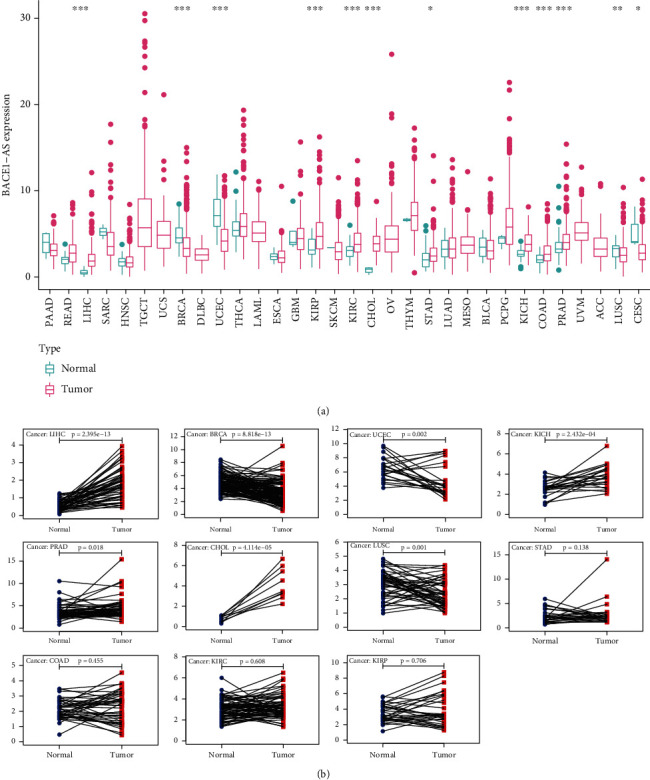
The abnormal expression level of BACE1-AS in 32 human tumors. (a) The expression level of BACE1-AS in pan-carcinomas from TCGA database. (b) Pairing differential analysis of BACE1-AS in tumors. ^∗^*P* < 0.05, ^∗∗^*P* < 0.01, and ^∗∗∗^*P* < 0.001.

The pan-cancer data of 32 primary tumors are described in Table 1. Based on the analysis of 10,443 samples from TCGA database, we found that BACE1-AS expression levels were upregulated across most cancer types including LIHC, KIRP, KIRC, CHOL, STAD, KICH, COAD, and PRAD. In contrast, BACE1-AS expression levels in BRCA, UCEC, LUSC, and CESC were higher in normal samples than in tumors (Figure 1(a)). Further analysis was performed on tumors with more than 5 pairs of tumor and adjacent normal tissues. The results of paired difference analysis indicated that BACE1-AS expression levels were higher in LIHC, CHOL, KICH, and PRAD tumor tissues while downregulated in BRCA, UCEC, and LUSC (Figure 1(b)).

### 3.2. The Prognostic Value of BACE1-AS in Pan-Cancer

**Figure 2:**
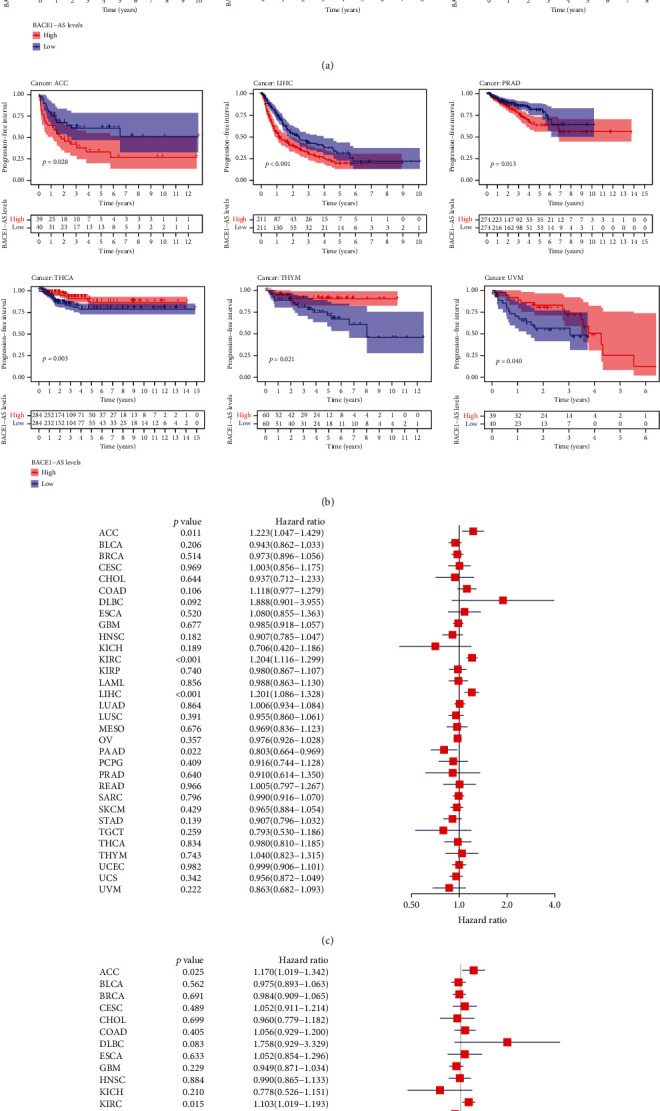
Prognostic value of BACE1-AS in different tumors. (a, b) Kaplan-Meier curves estimate the OS and PFI differences in pan-cancer. (c, d) OS and PFI differences among pan-cancer analyzed by univariate regression analysis. Only survival curves with significant differences (*P* < 0.05) were shown.

The correlations between BACE1-AS expression levels and the prognosis of different cancers are depicted in Figure 2 and Supplementary Figure 1. Patients with downregulated BACE1-AS expression in ACC, COAD, KIRC, and LIHC had longer overall survival (OS), while the opposite was detected in PAAD and UVM patients (Figure 2(a)). The PFI analysis (Figure 2(b)) found that longer progression-free intervals were associated with lower expression of BACE1-AS in ACC, LIHC, and PRAD. The DFI results (Supplementary Figure 1a) indicated that highly expressed BACE1-AS correlated negatively with DFI in LIHC and HNSC, while the opposite results were found in PCPG and THCA. High BACE1-AS expression predicted poorer disease-specific survival (DSS) in KIRC and LIHC but longer DSS times in KICH, PAAD, and UVM (Supplementary Figure 1b). A univariate Cox regression analysis indicated that BACE1-AS is a protective factor in PAAD and KICH but is a risk factor in ACC, COAD, LIHC, and KIRC (Figures 2(c) and 2(d), Supplementary Figures 1c and 1d).

### 3.3. Clinical Characteristics of BACE1-AS in Pan-Cancer

**Figure 3:**
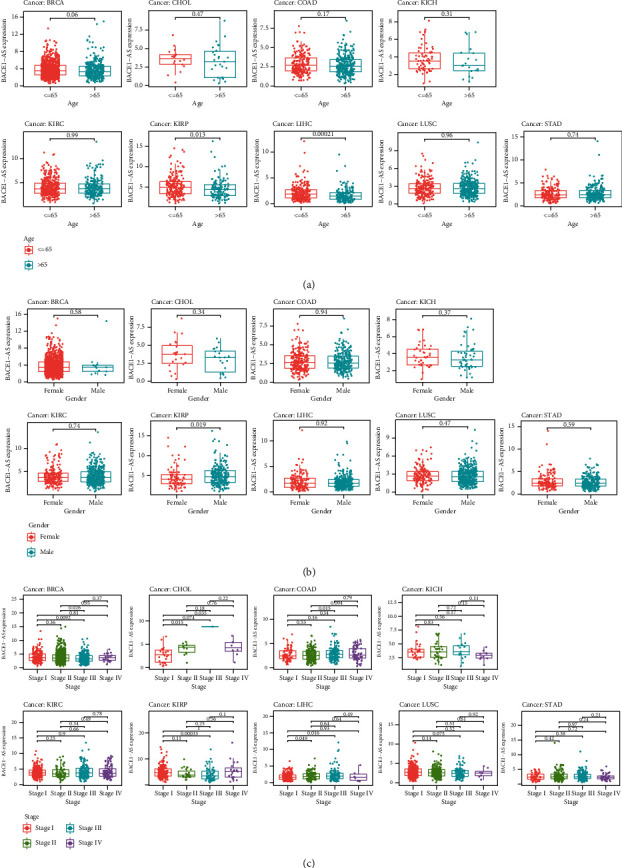
Correlation between the expression level of BACE1-AS and clinical characteristics. (a) Relationship between expression level of BACE1-AS and age. (b) Correlation analysis between expression level of BACE1-AS and gender. (c) The expression level of BACE1-AS in different clinical stages among different tumors.

The correlations between different clinical characteristics and BACE1-AS expression were further analyzed in 12 kinds of tumors with abnormal BACE1-AS expression. Considering that only one gender of PRAD, UCEC, and CESC was included in this study, no further analysis of the clinical characteristics of these three tumors was conducted. The results indicated that BACE1-AS expression levels in KIRP and LIHC (Figure 3(a)) were higher in patients younger than 65 years old (*P* < 0.05). BACE1-AS expression in male patients (Figure 3(b)) was higher than that in female patients only in KIRP (*P* < 0.05). In the other types of tumors, BACE1-AS expression was not significantly different between the genders. BACE1-AS expression levels were correlated with tumor stage in BRCA, KIRP, CHOL, COAD, and LIHC (Figure 3(c)). Generally, the higher the stage, the higher the expression level of BACE1-AS.

### 3.4. Mutation Analysis of BACE1-AS in Pan-Cancer

**Figure 4:**
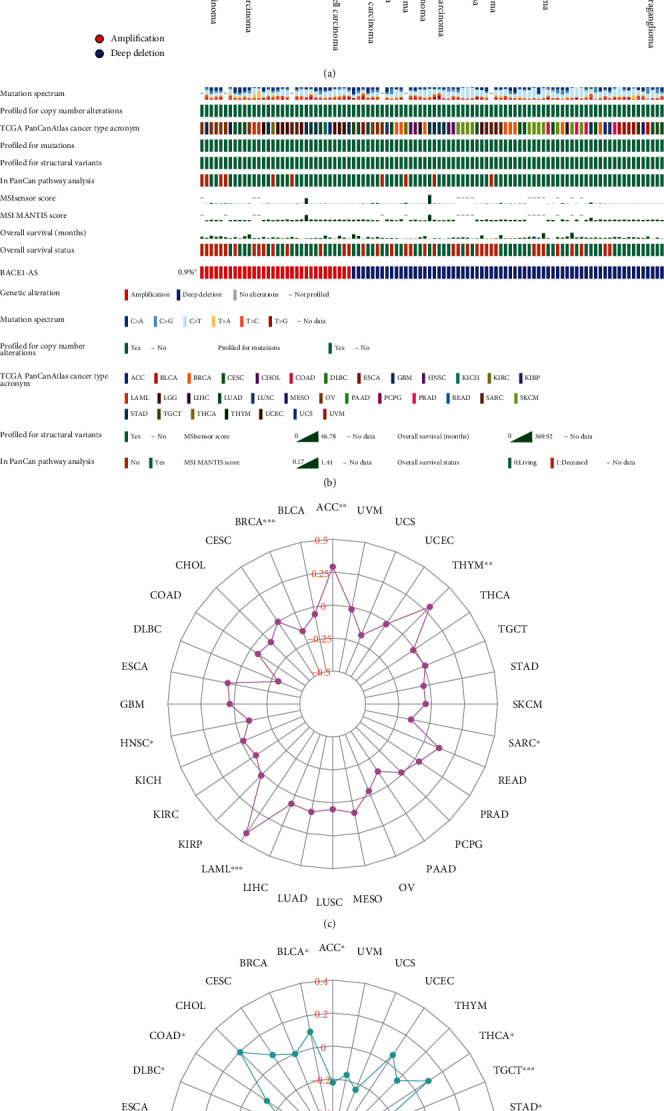
(a) Mutation characteristics of BACE1-AS in different carcinomas by using cBioPortal. (b) Detailed mutation information of BACE1-AS in mutated samples. (c) Correlation analysis of the expression level of TMB and BACE1-AS. (d) Correlation analysis of the expression level of MSI and BACE1-AS. ^∗^*P* < 0.05, ^∗∗^*P* < 0.01, and ^∗∗∗^*P* < 0.001.

Figures 4(a) and 4(b) depict the BACE1-AS mutation results from TCGA cancer database following cBioPortal online analysis. The results revealed that the mutation forms of BACE1-AS in tumors were mainly amplification and deep deletion and that the mutation rate was highly focused in UVM and UCS. The correlation (Spearman) between BACE1-AS expression and tumor mutational burden (TMB) (Figure 4(c)) was significant in ACC, BRCA, LAML, SARC, HNSC, and THYM. Also, microsatellite instability (MSI) (Figure 4(d)) was significantly associated with BACE1-AS expression levels in BLCA, ACC, COAD, DLBC, KIRC, LUAD, LUSC, PRAD, STAD, TGCT, and THCA, all *P* < 0.05. The correlation between BACE1-AS expression and tumor mutation load is shown in Table 2.

### 3.5. Correlation Analysis of BACE1-AS Expression and TME

**Figure 5:**
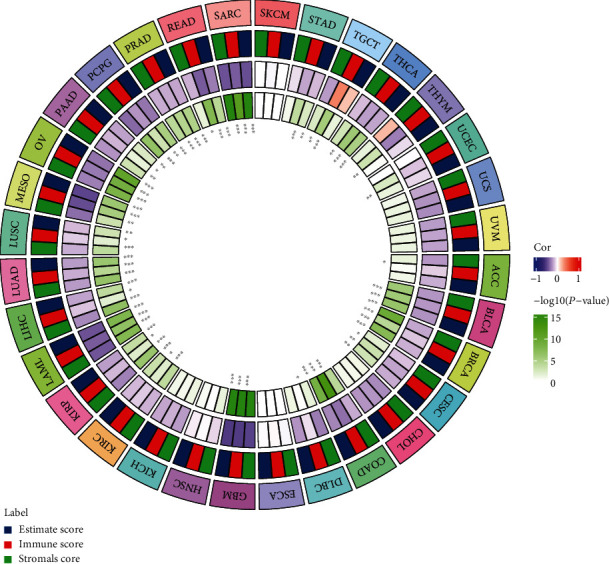
The correlation between the expression level of BACE1-AS and TME in pan-cancers. Association analysis of BACE1-AS with stromal score, immune score, and estimate score. ^∗^*P* < 0.05, ^∗∗^*P* < 0.01, and ^∗∗∗^*P* < 0.001.

We examined the correlation between BACE1-AS and TME (Figure 5) to evaluate the role of the BACE1-AS in tumor immunity. Accordingly, the tumors showing a significant correlation between BACE1-AS and immune scores in pan-cancers in TCGA database were BLCA, BRCA, CESC, COAD, GBM, KIRC, KIRP, LAML, LIHC, LUAD, LUSC, MESO, OV, PAAD, PCPG, PRAD, READ, SARC, STAD, TGCT, THCA, THYM, and UCEC (all *P* < 0.05; Supplementary Figure 2). Generally, BACE1-AS expression levels were inversely correlated with immune scores, except for TGCT.

The correlations between BACE1-AS expression and stromal scores and estimate score are shown in Supplementary Figures 3 and 4. The combined analysis of immune and stromal scores is denoted by the estimate scores. Both stromal and estimate scores were negatively correlated with BACE1-AS expression levels in tumors. The correlation between BACE1-AS and TME in GBM was the most significant, with BACE1-AS having the highest correlation coefficients with immune scores (*R* = −0.53, *P* < 0.05), stromal scores (*R* = −0.51, *P* < 0.05), and estimate scores (*R* = −0.54, *P* < 0.05).

### 3.6. Correlation Analysis of BACE1-AS with Immune Cell Infiltration in Pan-Cancer

**Figure 6:**
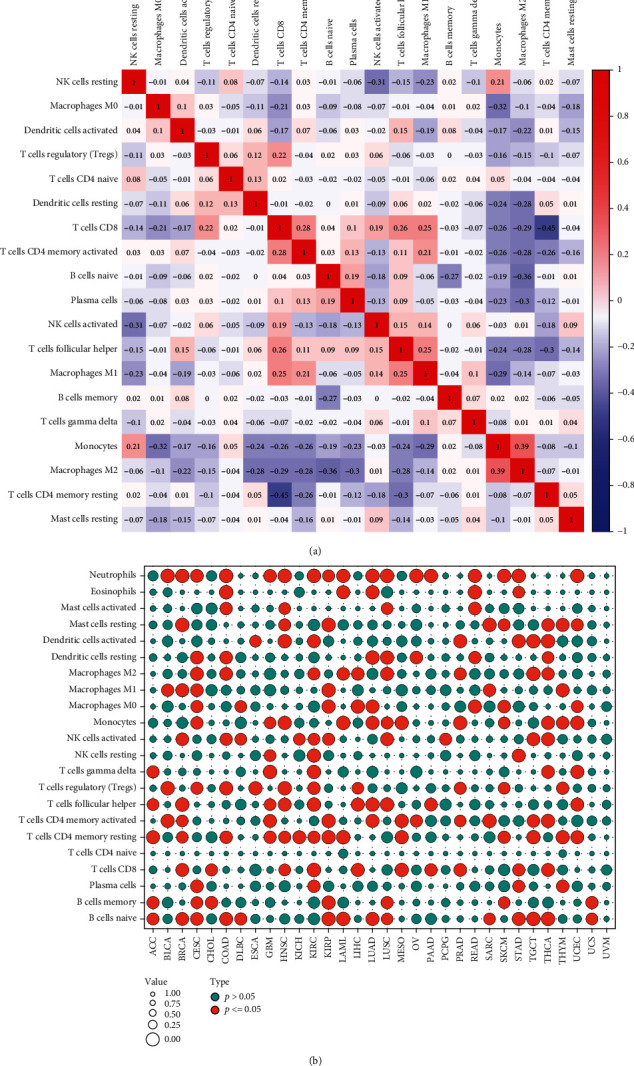
The correlation between the expression level of BACE1-AS and immune cell infiltration analysis in pan-cancers. (a) Correlation analysis between immune cells. The blue circle shows a negative correlation between the two genes, while the red circle shows a significant positive correlation. (b) Correlation of BACE1-AS expression with immune cells in pan-cancer. The red circles indicated BACE1-AS expression was significantly correlated with immune cells, while the blue circles indicated no correlation.

**Figure 7:**
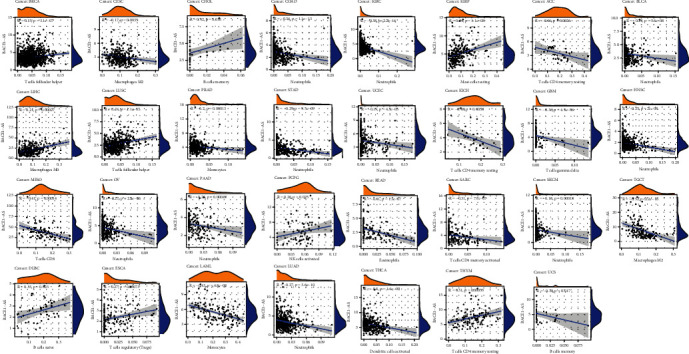
Correlation of BACE1-AS expression with immune cells in pan-cancer. Only the most closely related tumor infiltrating cells were exported in the figure.

Figure 6(a) indicates that the strongest negative correlation is between T cells CD4 memory resting and T cells CD8 (*R* = −0.45, *P* < 0.05), while monocytes and macrophages M2 have the most significant positive correlation (*R* = 0.39, *P* < 0.05). The correlation analysis between 22 immune cell types and pan-cancer as based on the TIMER database is illustrated in Figures 6(b) and 7. Neutrophils, monocytes, regulatory T cells (Tregs), follicular T cells, activated memory CD4 T cells, resting memory CD4 T cells, and naïve B cells were significantly associated with most tumors. Of all the infiltrating immune cells, neutrophils had the highest correlation in 10 tumor types including BLCA, COAD, HNSC, PAAD, SKCM, STAD, LUAD, OV, KIRC, and UCEC, indicating that the higher the BACE1-AS level, the lower the neutrophil content. Resting memory CD4 T cells were negatively correlated with ACC and KICH and positively correlated with THYM. Memory B cells were positively correlated with CHOL and negatively correlated with UCS. The highest correlations, both positive, with BRCA and LUSC were with follicular helper T cells. CESC and TGCT were most significantly negatively correlated with M2 macrophages, while monocytes were most significantly negatively correlated with LAML and PRAD. The strongest correlations with MESO, DLBC, THCA, READ, LIHC, KIRP, PCPG, SARC, GBM, and ESCA were with CD8 T cells, naïve B cells, activated dendritic cells, eosinophils, M0 macrophages, resting mast cells, activated natural killer (NK) cells, activated memory CD4 T cells, gamma delta T cells, and Tregs, respectively.

### 3.7. Correlation of BACE1-AS Expression with Immune Marker Sets

Immune checkpoints are one of the most promising targets for cancer therapy. They protect the immune system by preventing T cell overactivation from causing damage to the body. The upregulation of these immune marker sets can induce immune escape to inhibit the antitumor response of the immune system. Thus, we analyzed the relationship between 46 immune checkpoint genes and pan-cancer.

**Figure 8:**
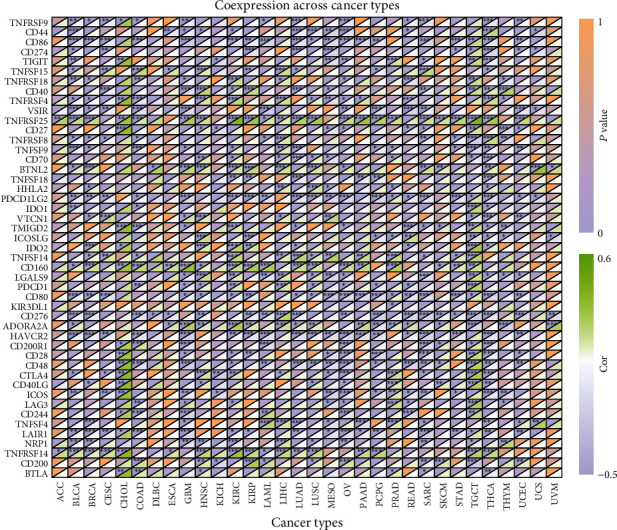
Heat map of correlation analysis between BACE1-AS expression and immune marker sets. ^∗^*P* < 0.05, ^∗∗^*P* < 0.01, and ^∗∗∗^*P* < 0.001.

We found that THCA, TGCT, OV, GBM, and KIRC were the top five tumors significantly correlated with most immune marker genes (Figure 8). BACE1-AS expression had strong positive correlations with most of the immunomarker genes in TGCT, CHOL, HNSC, KIRC, and LIHC. Further, *TNFRSF25*, *PDCD1LG2*, *CD276*, *CD80*, *HAVCR2*, *LAIR1*, *CD160*, *TNFRSF14*, *ADORA2A*, and *CD48* were significantly associated with BACE1-AS expression in more than half of the tumor types. Notably, *KIR3DL1* was the gene negatively correlated with BACE1-AS expression only in PRAD (*R* = −0.10, *P* < 0.05). ACC was significantly correlated with *TNFRSF25* (*R* = 0.34, *P* < 0.05) and *TNFRSF14* (*R* = 0.22, *P* < 0.05) only. Finally, DLBC was significantly correlated with *CD276* (*R* = −0.34, *P* < 0.05) and *BTNL2* (*R* = 0.33, *P* < 0.05).

### 3.8. Gene Set Enrichment Analysis in the Low- and High-Expression BACE1-AS Groups

We applied Gene Ontology (GO) and Kyoto Encyclopedia of Genes and Genomes (KEGG) enrichment analysis on different BACE1-AS expression levels in pan-cancer. We found that KEGG function between the high- and low-expression BACE1-AS groups was significantly different in 15 types of tumors and was significantly different in GO enrichment across all tumor types.

**Figure 9:**
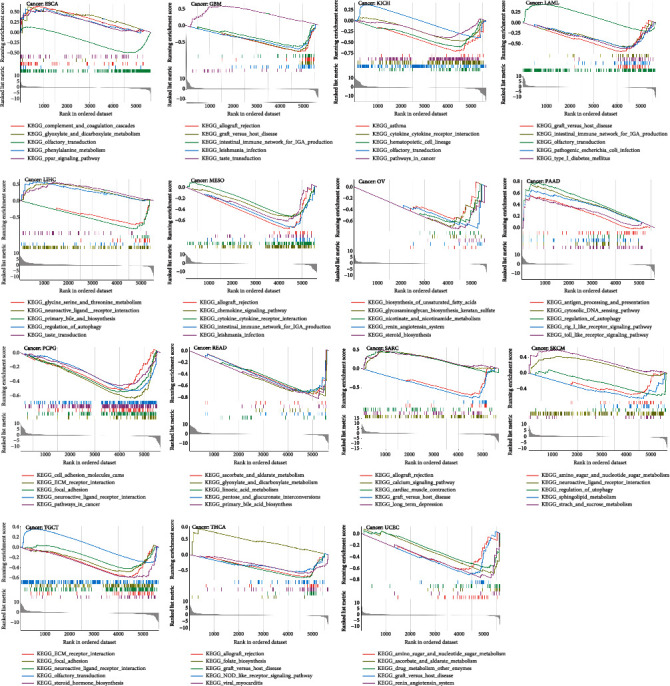
KEGG analysis of high and low expression of BACE1-AS in pan-cancer. Each of the different colored lines represents a specific gene set. The upregulated genes were showed at the left side near the origin, while the downregulated gene set was at the right side. Only *P* value < 0.05 was considered significant.

BACE1-AS expression mainly affected pathways including complement and coagulation cascades, RRAR signaling pathway, intestinal immune network for immunoglobulin A (IgA) production, cancer pathways, autophagy regulation, cytokine-cytokine receptor interaction, and antigen processing and presentation, which are mainly related to tumorigenesis and immune activity (Figure 9). The result of the GO analysis is shown in Supplementary Figure 5.

### 3.9. Expression Characteristics and Role of BACE1-AS in Hepatocellular Carcinoma (HCC)

**Figure 10:**
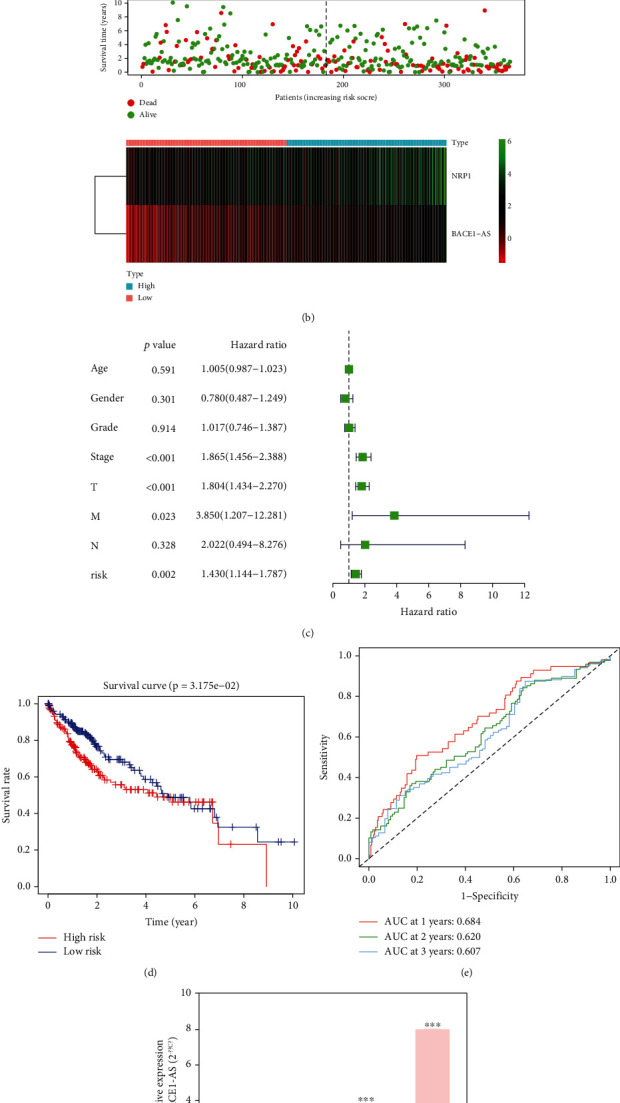
Validation of the role of BACE1-AS in hepatocellular carcinoma. (a) The combined effect of BACE1-AS and NRP1 on the prognosis of hepatocellular carcinoma. (b) Analysis of survival status and gene expression in high- and low-risk patients. (c) Independent prognostic analysis of the 2-gene signature model risk score. (d) Prognosis difference between high- and low-risk groups of HCC patients. (e) Area under the curve of the risk model at 1, 2, and 3 years. (f) Expression level of BACE1-AS in normal liver cells and liver cancer cells. (g) Evaluation of the effect of risk model on drug sensitivity.

The role of BACE1-AS in tumorigenesis and its relationship with immunity in pan-cancer cannot be ignored by the above analysis. However, experimental evidence is lacking. We further validated the role of BACE1-AS in HCC at cellular levels using quantitative real-time polymerase chain reaction (qRT-PCR).  Figure 10(f) shows that BACE1-AS expression levels were significantly increased in the Hep3B, HLE, and HLF cell lines compared to the normal liver cell line LO2 (*P* < 0.05).

19 immune checkpoint genes (*CD276*, *TNFRSF14*, *ADORA2A*, *TNFSF15*, *BTNL2*, *TNFRSF4*, *PDCD1*, *TNFSF4*, *NRP1*, *TNFRSF8*, *TNFRSF25*, *LGALS9*, *HHLA2*, *TNFSF14*, *TNFSF9*, *TNFRSF18*, *VTCN1*, *CD70*, and *CTLA4*) were identified significantly associated with BACE1-AS expression (Figure 8). To further verify the interaction between BACE1-AS and immune checkpoint genes in tumors, a prognostic model using Cox regression analysis was established using BACE1-AS and *NRP1*, an immune checkpoint gene coexpressed with BACE1-AS.  Figure 10(c) indicates that the model can predict the prognosis of HCC patients independently of other clinical factors. The area under the time-dependent curve (AUC) of 1-, 2-, and 3-year survival in the risk model was 0.684, 0.620, and 0.607 (Figure 10(e)). The patients were divided into risk groups according to their median risk score. In the low-risk group, more patients survived with higher survival rates, and BACE1-AS and NRP1 were more highly expressed (Figures 10(b) and 10(d)). By analyzing the effect of *NRP1* and BACE1-AS expression levels on patient survival, it was found that patients with high expression levels of both NRP1 and BACE1-AS had the lowest survival rate. In contrast, survival rate was the highest when both NRP1B and BACE1-As expression levels were low (Figure 10(a)). Moreover, this two-gene signature model can significantly predict the sensitivity of common chemotherapeutic drugs (Figure 10(g)); for example, higher IC50s and lower sensitivities were predicted in axitinib, docetaxel, erlotinib, methotrexate, sorafenib, and sunitinib in high-risk patients, while a lower IC50 and higher sensitivity of doxorubicin were predicted in high-risk patients (all *P* < 0.05).

## 4. Discussion

BACE1-AS, a conserved noncoding antisense transcript for BACE1 that can bind to BACE1 mRNA to improve its stability, was reported to be upregulated and a potential biomarker for Alzheimer's disease [12, 13]. Notably, the expression level of BACE1-AS has been reported to play an important role in the progression of gastric cancer [14]. Moreover, anisomycin can inhibit the proliferation and invasion of ovarian cancer stem cells by increasing BACE1-AS levels [21]. Using bioinformatics, Nie et al. identified BACE1-AS as a poor prognostic factor for HCC [22]. Here, we found that BACE1-AS was highly expressed in most tumor types, and its abnormal expression affected the survival times of some tumors. Moreover, from our qRT-PCR experiment, the expression levels of BACE1-AS in three HCC cell lines were higher than in LO2. Our results are highly consistent with previous studies. Altogether, the aforementioned direct or indirect data suggest that the role of BACE1-AS in tumors cannot be ignored.

With the development of molecular biology, great progress has been made in the study of tumor etiology and pathogenesis. However, the occurrence and development of tumors are extremely complex with multiple factors and multiple steps. More specifically, the occurrence of tumors is not simply the result of a single gene mutation; it is a long-term, phasic, accumulative process involving multiple gene mutations [1, 4]. We reported that TMB was significant in ACC, BRCA, LAML, SARC, HNSC, and THYM and that MSI was significantly associated with BACE1-AS expression levels in 11 types of tumors. These findings indicate the potential mechanisms of BACE1-AS in mediating tumorigenesis from the perspective of tumor mutation.

TME, a fundamental direction in tumor research, plays an important role in the diagnosis, prevention, and prognosis of tumors; it is of great significance in understanding tumor occurrence, development, and metastasis [23]. Presently, it is believed that the body's immune surveillance system plays an important role in preventing the occurrence of tumors; thus, the occurrence of tumors may result from the loss of immune surveillance [24, 25]. Our study is the first to analyze the relationship between BACE1-AS expression and tumor immunity of pan-cancer, including immune microenvironment, infiltrating immune cells, and immune checkpoint targets. BACE1-AS expression was correlated with immune scores in 23 types of tumors: it was negatively correlated with 22 types and positively correlated with TCGT. The higher the expression of BACE1-AS, the smaller the proportions of stromal and estimate scores in pan-cancer. We know that the higher the three immune scores, the higher the content of immune cells in the tumor sample and the lower the purity of the tumor cells. In other words, tumor cells are fewer when BACE1-AS expression is low. This finding highly corresponds with the higher expression of BACE1-AS in LIHC, KIRP, KIRC, CHOL, STAD, KICH, COAD, and PRAD compared to that in normal tissues. The analysis of immune components in the TME can help increase the understanding of how immune composition and immune status can affect cancer cells and cancer treatment. To some extent, the occurrence of tumors is an abnormal inflammatory response. Tumor cells recruit inflammatory cells to reach tumor tissues by producing various inflammatory factors, such as growth factors, cytokines, and chemokines [26, 27].

Immune infiltrating cells in tumor tissues can change the metabolism and function of tumor cells and promote tumor immunosuppression and immune escape [28]. We analyzed 22 types of effector cells involved in the tumor immune response in pan-cancer. We found that nine immune-associated cells were significantly associated with BACE1-AS expression levels in more than 10 types of tumors (*P* < 0.05). However, the expression levels of BACE1-AS in UVM were not significantly correlated with these immune cells. Neutrophils were significantly negatively correlated with BACE1-AS expression in the most common tumor types. Notably, infiltrating neutrophils may promote tumor-associated inflammation or, through the expression of antitumor and cytotoxic mediators, inhibit tumor growth [29]. This characteristic of neutrophils may explain why most tumors are more aggressive and have poorer prognosis when BACE1-AS expression is upregulated. NK cells and BACE1-AS expression levels were negatively correlated in four tumor types and positively correlated in six. NK cells play a vital role in antitumor immunity by directly recognizing and killing tumor cells. Even tumor cells that develop strategies to evade recognition by CD8+ T cells are attacked by NK cells [30]. We also found that BACE1-AS expression levels were negatively correlated with Tregs in two types of tumors and positively correlated in eight types, indicating that BACE1-AS may be involved in the regulation of tumor immunity. Macrophages can play an immunosuppressive role via the PI3K*γ* signaling pathway. Based on their phenotypes and functions, macrophages can be divided into classically activated macrophages (M1 type) and alternatively activated macrophages (M2 type), also known as tumor-associated macrophages (TAMS). TAMs can prevent T cells from attacking tumor cells, secrete growth factors to nourish tumor cells, promote the formation of blood vessels in tumor tissues, and further promote the metastasis and proliferation of tumor cells [31, 32]. We found that M2 macrophages were negatively correlated with BACE1-AS in TGCT, LIHC, LUCC, COAD, PRAD, and KIRC and positively correlated with BACE1-AS in THCA, CESC, and LAML. These results suggest that BACE1-AS may play an immunosuppressive role in cancer by promoting the presentation of tumor antigens.

Immune checkpoint molecules, such as CTLA4, NRP1, TNFSF15, CD44, and BTLA, are present in the TME or on the surface of tumor cells, and they negatively regulate T cell activation. Under normal conditions, these molecules mainly maintain T cell homeostasis, but during tumor evolution, these molecules can help tumor cells escape the immune response [33, 34]. CTLA4 and PD-L1 (CD274) are two relatively mature immune checkpoint molecules. CTLA4 and BACE1-AS expression was significantly positively correlated in seven types of tumors and negatively correlated in six, while CD274 was significantly correlated with BACE1-AS expression in 10 types of tumors. This data not only further reveal the potential association between BACE1-AS and tumor immunity but also suggest that we can further experimentally verify the effectiveness of immunosuppressive therapies in relevant cancer types. TNFRSF25, a T cell costimulatory molecule, which we found to be significantly associated with BACE1-AS expression among 23 types of tumors, is correlated with the pathogenesis of liver cancer, pancreatic cancer, breast cancer, colon cancer, and leukemia and activates the NF-*κ*B and MARK signaling pathways [35–39]. Thus, TNFRSF25 may be a key immune checkpoint molecule mediating the effect of BACE1-AS on tumor progression, and further study on the association between the two may help to develop new therapeutic targets. The results of the prognostic model on HCC prognosis and chemosensitivity as constructed using BACE1-AS and NRP1 further confirm the close relationship between BACE1-AS and tumor immunity in tumor progression.

We found that BACE1-AS expression affects pathways related to tumorigenesis and immune activity. These pathways include the complement and coagulation cascades, RRAR signaling pathway, intestinal immune network for IgA production, cancer pathways, autophagy regulation, cytokine-cytokine receptor interaction, and antigen processing and presentation. These results account for the association between BACE1-AS expression and tumor immunity from the perspective of internal mechanisms, but further experimental investigation is needed for verification.

Overall, our results suggest that BACE1-AS is abnormally expressed in most tumor types, and its expression level is correlated with clinical features, prognosis, TMB, and MSI. Moreover, BACE1-AS expression may affect the TME, the content of infiltrating immune cells, the immune molecular checkpoints, and the immune activity-related pathways. Altogether, our data indicate that BACE1-AS can be a potential immunotherapy target in improving cancer patient outcomes.

## Figures and Tables

**Table 1:** Clinical features of pan-cancer from TCGA database.

Abbreviation	Tumor type	Normal	Tumor	Female	Male	Average age
ACC	Adrenocortical carcinoma	0	79	48	31	46.70
BLCA	Bladder urothelial carcinoma	19	408	114	313	68.17
BRCA	Breast invasive carcinoma	113	1097	1197	13	58.21
CESC	Cervical squamous cell carcinoma and endocervical adenocarcinoma	3	306	309	0	48.30
CHOL	Cholangiocarcinoma	9	36	23	22	64.56
COAD	Colon adenocarcinoma	35	461	234	262	67.51
DLBC	Lymphoid neoplasm diffuse large B cell lymphoma	0	48	26	22	56.27
ESCA	Esophageal carcinoma	11	162	29	144	63.82
GBM	Glioblastoma multiforme	5	161	62	104	55.06
HNSC	Head and neck squamous cell carcinoma	44	502	152	394	60.87
KICH	Kidney chromophobe	24	65	38	51	52.60
KIRC	Kidney renal clear cell carcinoma	72	531	217	386	61.54
KIRP	Kidney renal papillary cell carcinoma	32	289	88	233	61.35
LAML	Acute myeloid leukemia	0	151	68	83	50.00
LIHC	Liver hepatocellular carcinoma	50	374	141	233	59.79
LUAD	Lung adenocarcinoma	59	515	309	265	65.44
LUSC	Lung squamous cell carcinoma	49	501	143	407	67.50
MESO	Mesothelioma	0	86	15	71	62.95
OV	Ovarian serous cystadenocarcinoma	0	379	379	0	60.16
PAAD	Pancreatic adenocarcinoma	4	178	79	103	65.10
PCPG	Pheochromocytoma and paraganglioma	3	183	103	83	47.32
PRAD	Prostate adenocarcinoma	52	496	0	548	60.87
READ	Rectum adenocarcinoma	10	166	84	92	64.19
SARC	Sarcoma	2	263	145	121	61.12
SKCM	Skin cutaneous melanoma	1	471	179	293	58.15
STAD	Stomach adenocarcinoma	32	375	161	246	66.32
TGCT	Testicular germ cell tumors	0	139	0	139	31.87
THCA	Thyroid carcinoma	58	510	414	154	47.02
THYM	Thymoma	2	119	60	61	57.91
UCEC	Uterine corpus endometrial carcinoma	35	532	567	0	63.68
UCS	Uterine carcinosarcoma	0	56	56	0	69.77
UVM	Uveal melanoma	0	80	35	45	61.65

**Table 2:** Correlation between BACE1-AS expression and tumor mutation load.

Cancer type	TMB		MSI	
	*R*	*P* value	*R*	*P* value
ACC	0.29	<0.01^∗∗^	-0.22	<0.05^∗^
BLCA	-0.06	0.26	0.10	<0.05^∗^
BRCA	-0.15	<0.001^∗∗∗^	0.00	0.92
CESC	0.00	0.99	0.06	0.33
CHOL	-0.09	0.61	0.19	0.25
COAD	-0.07	0.18	-0.12	<0.05^∗^
DLBC	-0.30	0.07	-0.30	<0.05^∗^
ESCA	0.06	0.44	0.08	0.35
GBM	0.03	0.71	0.09	0.27
HNSC	-0.10	<0.05^∗^	0.07	0.13
KICH	-0.02	0.90	0.07	0.56
KIRC	-0.05	0.37	-0.15	<0.01^∗∗^
KIRP	0.02	0.77	-0.03	0.58
LAML	0.43	<0.001^∗∗∗^	0.19	0.11
LIHC	0.07	0.19	0.06	0.24
LUAD	0.08	0.06	0.30	<0.001^∗∗∗^
LUSC	0.05	0.28	0.26	<0.001^∗∗∗^
MESO	0.09	0.42	-0.01	0.96
OV	-0.03	0.57	0.10	0.11
PAAD	-0.13	0.10	-0.10	0.21
PCPG	-0.01	0.85	0.08	0.29
PRAD	0.04	0.42	0.12	<0.01^∗∗^
READ	0.12	0.16	0.14	0.09
SARC	-0.15	<0.05^∗^	0.00	0.95
SKCM	-0.05	0.33	0.01	0.83
STAD	-0.05	0.36	0.11	<0.05^∗^
TGCT	0.01	0.92	-0.30	<0.001^∗∗∗^
THCA	-0.02	0.72	0.10	<0.05^∗^
THYM	0.29	<0.01^∗∗^	-0.05	0.60
UCEC	-0.02	0.61	0.06	0.20
UCS	-0.19	0.17	-0.24	0.08
UVM	-0.02	0.87	-0.17	0.14

## Data Availability

The datasets presented in this study can be found in online repositories. The names of the repository/repositories and accession number(s) can be found in the article.

## Supplementary Materials

Supplementary 1 Supplementary Figure 1: prognostic value of BACE1-AS in different tumors. (a, b) Kaplan-Meier curves estimate the DFI and DSS differences in pan-cancer. (c, d) DFI and DSS differences among pan-cancer analyzed by univariate regression analysis. Only survival curves with significant differences (*P* < 0.05) were shown.

Supplementary 2 Supplementary Figure 2: correlation difference of BACE1-AS expression and immune score in tumors with *P* < 0.05 was shown. ^∗^*P* < 0.05, ^∗∗^*P* < 0.01, and ^∗∗∗^*P* < 0.001.

Supplementary 3 Supplementary Figure 3: the correlation between BACE1-AS expression and stromal score. The stromal scores were negatively correlated with BACE1-AS expression levels in tumors with *P* value < 0.05 shown.

Supplementary 4 Supplementary Figure 4: relationship between expression level of BACE1-AS and tumor estimate score. The figure shows a significant correlation between the types of tumors. Tumor types with a significant negative correlation with BACE1-AS expression were shown in the figure.

Supplementary 5 Supplementary Figure 5: GO analysis reveals the molecular functions related to the expression level of BACE1-AS. Different colored curves represent different functions. The peak value of the curve at the top and in the part of positive gene correlation indicates that molecular functions here were positively correlated with BACE1-AS expression.
