# Supplementary figures and images for: Identification and Validation of the lncRNA BACE1-AS as Immune-Related Influencing Factor in Tumorigenesis following Pan-Carcinoma Analysis
Source: J Immunol Res. 2021 Dec 8;2021:1589864. doi: 10.1155/2021/1589864 (PMC8674649; doi:10.1155/2021/1589864)

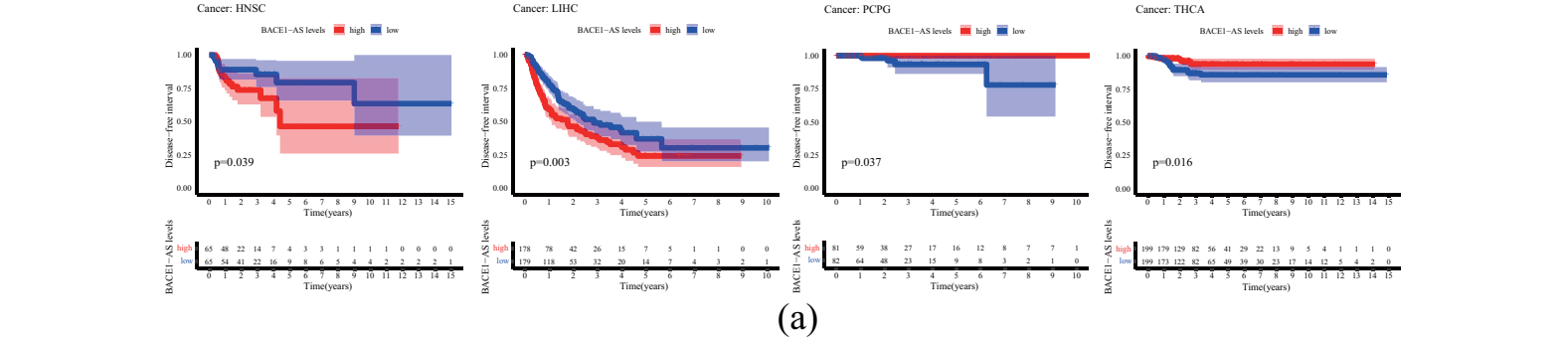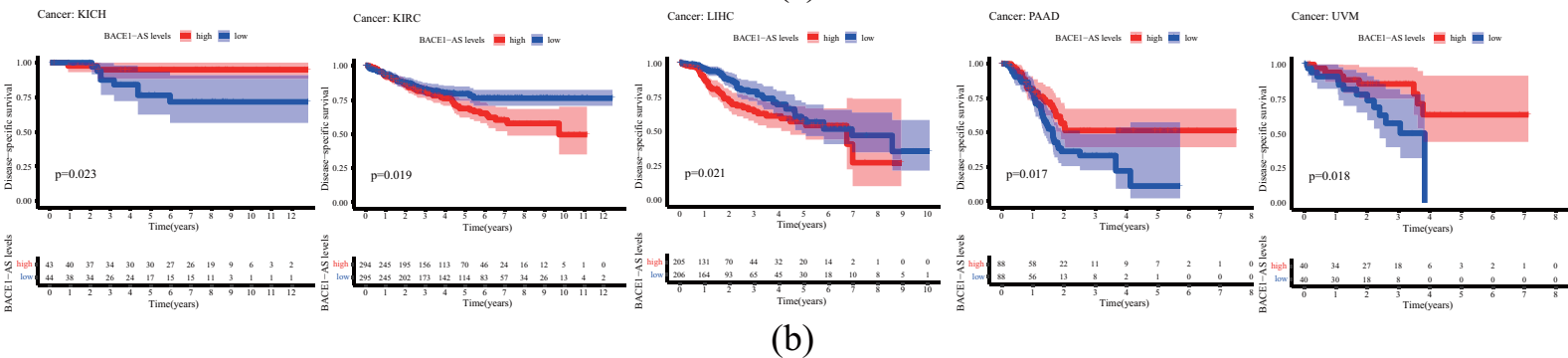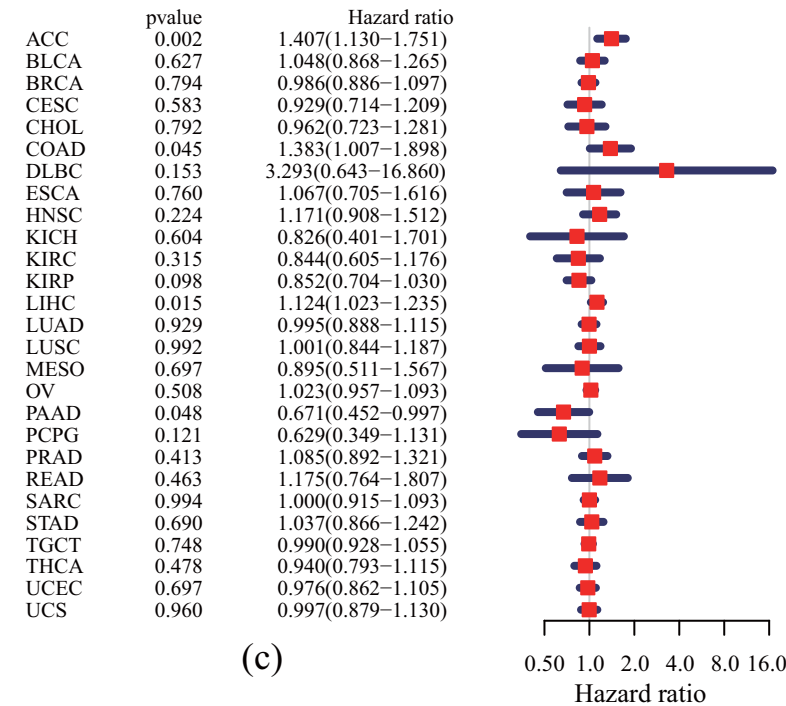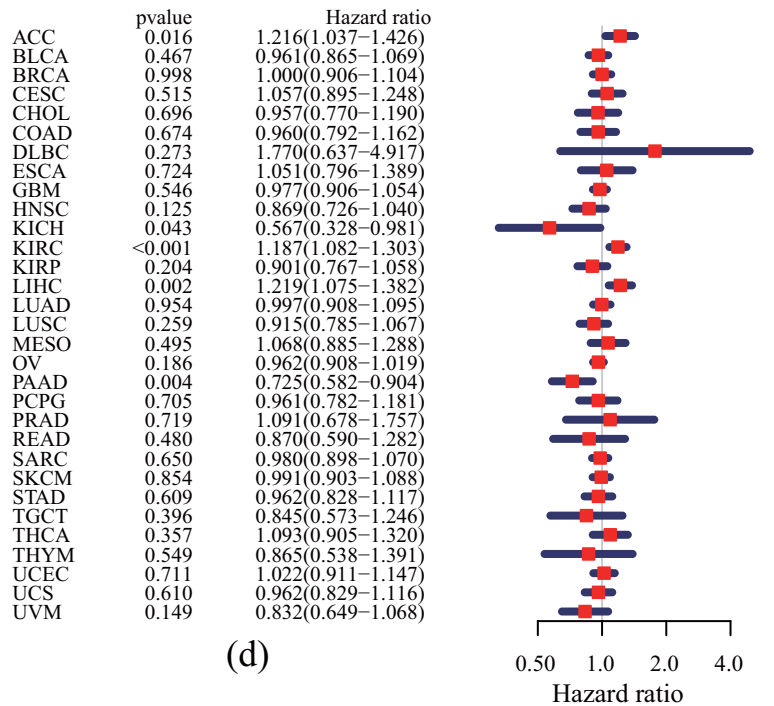

Supplement: Supplementary 1 — Supplementary Figure 1: prognostic value of BACE1-AS in different tumors. (a, b) Kaplan-Meier curves estimate the DFI and DSS differences in pan-cancer. (c, d) DFI and DSS differences among pan-cancer analyzed by univariate regression analysis. Only survival curves with significant differences (P < 0.05) were shown. [file 1589864.f1.pdf]

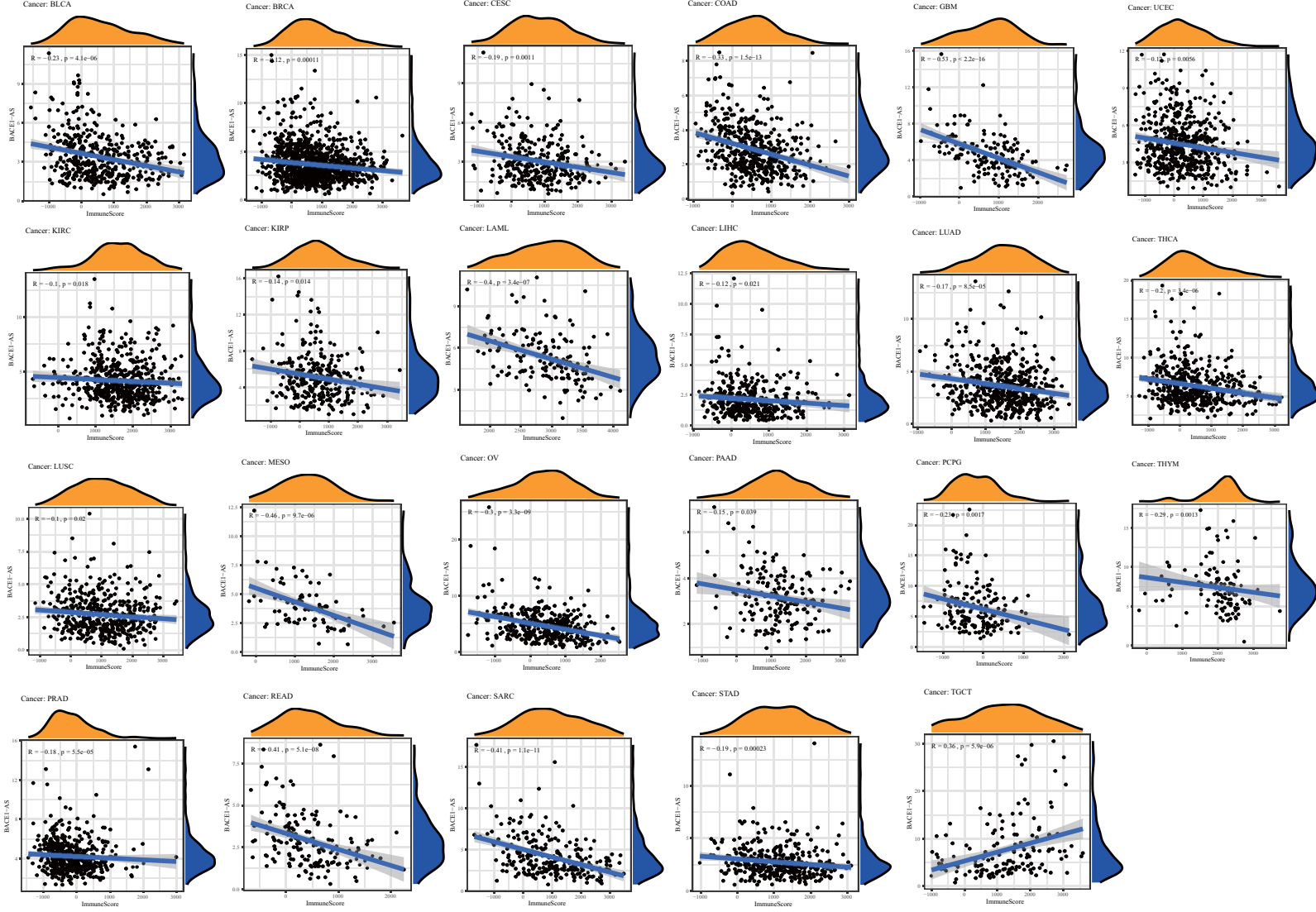

Supplement: Supplementary 2 — Supplementary Figure 2: correlation difference of BACE1-AS expression and immune score in tumors with P < 0.05 was shown. ∗P < 0.05, ∗∗P < 0.01, and ∗∗∗P < 0.001. [file 1589864.f2.pdf]

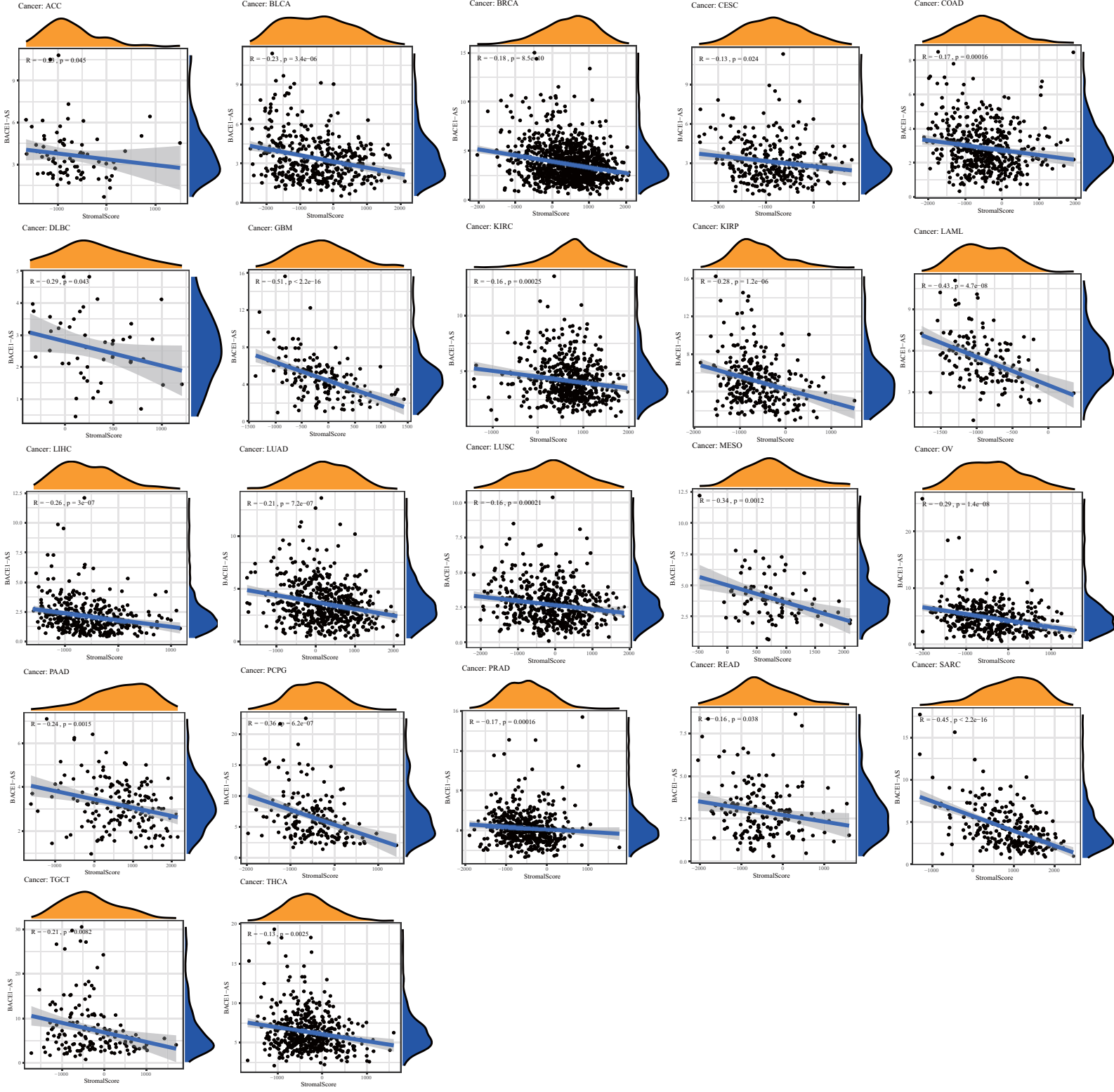

Supplement: Supplementary 3 — Supplementary Figure 3: the correlation between BACE1-AS expression and stromal score. The stromal scores were negatively correlated with BACE1-AS expression levels in tumors with P value < 0.05 shown. [file 1589864.f3.pdf]

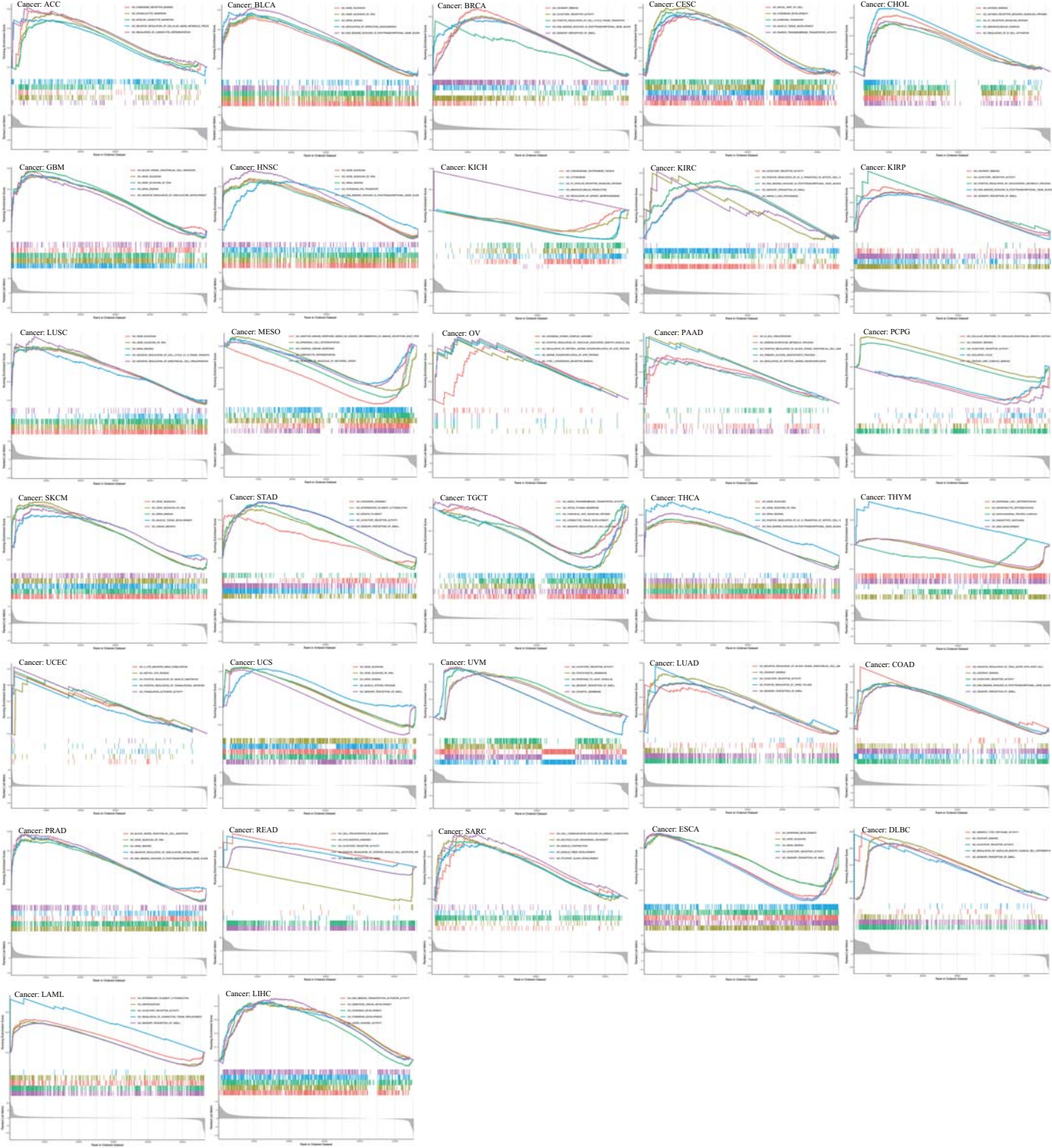

Supplement: Supplementary 5 — Supplementary Figure 5: GO analysis reveals the molecular functions related to the expression level of BACE1-AS. Different colored curves represent different functions. The peak value of the curve at the top and in the part of positive gene correlation indicates that molecular functions here were positively correlated with BACE1-AS expression. [file 1589864.f5.pdf]
