# Supplementary material for: Identification and Validation of the lncRNA BACE1-AS as Immune-Related Influencing Factor in Tumorigenesis following Pan-Carcinoma Analysis
Source: J Immunol Res. 2021 Dec 8;2021:1589864. doi: 10.1155/2021/1589864 (PMC8674649; doi:10.1155/2021/1589864)
Supplement: Supplementary 4 — Supplementary Figure 4: relationship between expression level of BACE1-AS and tumor estimate score. The figure shows a significant correlation between the types of tumors. Tumor types with a significant negative correlation with BACE1-AS expression were shown in the figure. [file 1589864.f4.pdf]

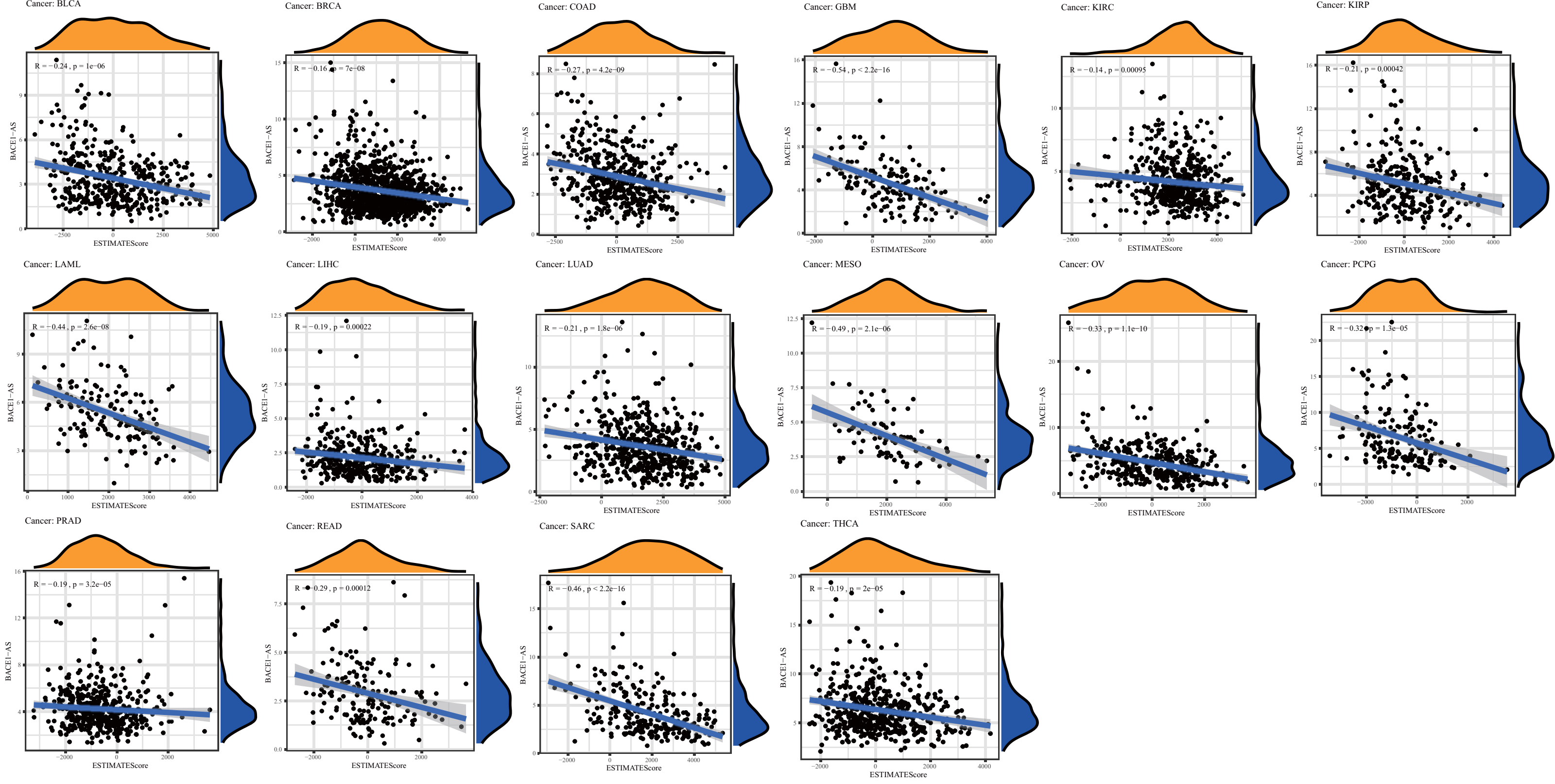
Cancer: BLCA
R = −0.24 , p = 1e−06
Cancer: BRCA
R = −0.16 , p = 7e−08
Cancer: COAD
R = −0.27 , p = 4.2e−09
Cancer: GBM
R = −0.54 , p < 2.2e−16
Cancer: KIRC
R = −0.14 , p = 0.00095
Cancer: KIRP
R = −0.21 , p = 0.00042
Cancer: LAML
R = −0.44 , p = 2.6e−08
Cancer: LIHC
R = −0.19 , p = 0.00022
Cancer: LUAD
R = −0.21 , p = 1.8e−06
Cancer: MESO
R = −0.49 , p = 2.1e−06
Cancer: OV
R = −0.33 , p = 1.1e−10
Cancer: PCPG
R = −0.32 , p = 1.3e−05
Cancer: PRAD
R = −0.19 , p = 3.2e−05
Cancer: READ
R = −0.29 , p = 0.00012
Cancer: SARC
R = −0.46 , p < 2.2e−16
Cancer: THCA
R = −0.19 , p = 2e−05
BACE1−AS
ESTIMATEScore
